# Isolation and Identification of Effective Probiotics on Drug-Resistant *Acinetobacter baumannii* Strains and Their Biofilms

**DOI:** 10.1155/2024/8570521

**Published:** 2024-02-26

**Authors:** Zahra Abbasi, Seyed Mahdi Ghasemi, Yasaman Ahmadi, Dariush Shokri

**Affiliations:** ^1^Department of Microbiology, Faculty of Biological Sciences and Technology, Shahid Ashrafi Esfahani University, Isfahan, Iran; ^2^Department of Microbiology, Kish International Branch of Islamic Azad University, Kish, Iran

## Abstract

**Introduction:**

This study aimed to identify, assess, and isolate strong lactobacilli demonstrating broad antibacterial and anti-biofilm activity against drug-resistant strains of *Acinetobacter baumannii*. Additionally, the mechanism of inhibition of these organisms was to be determined.

**Methods:**

Over a 6-month period (from December 2021 to June 2022), 53 clinical *A. baumannii* strains were collected from clinical samples. Twenty probiotic strains were isolated from local dairy products. Antibacterial activity of *Lactobacillus* strains' cell-free supernatant (CFS) was identified using the agar well diffusion method and the microbroth dilution test. Anti-biofilm effect was performed by the microtiter plate assay. The MTT assay was also used to look into the probiotics' cytotoxic effects on the L929 fibroblast cell line.

**Results:**

During the 6-month period, 53 clinical *A. baumannii* strains were obtained and identified. Out of 20 *lactobacillus* strains, the CFS of a *lactobacillus* strain (named L9) showed an inhibitory effect against all *A. baumannii* strains. Using the broth microdilution method, it was shown that the minimum inhibitory concentrations (MICs) and minimum bactericidal concentrations (MBCs) of CFS extracts of L9 strains against *A. baumannii* strains were both ¼ mg/mL. The result of the anti-biofilm showed that the selected probiotic could inhibit biofilm formation. The most common organic acid produced by all *Lactobacillus* strains, according to the HPLC method, was lactic acid, which was followed by acetic acid. The L929 fibroblast cell line was used in the cytotoxicity assay, which revealed that 100% of the cells in the L929 fibroblast cell line survived treatment with successive doses of CFSs for a full day.

**Conclusion:**

The probiotic strain isolated from local yogurt in this study showed potential anti-biofilm and antimicrobial properties against all drug-resistant *Acinetobacter* isolates. Given the increasing interest in probiotic microorganisms based on their high health benefits, further studies are recommended on the mechanisms of action between probiotics and *A. baumannii* strains to find new solutions for biological control and treatment of these infections without the use of antibiotics.

## 1. Introduction

Worldwide, the prevalence of multidrug-resistant (MDR) microorganisms is rising, presenting a major health concern to individuals [1, 2]. Hospital-acquired infections caused by MDR strains have resulted in increased treatment costs, mortality, and morbidity, thereby compromising patient safety. In response to the surge in MDR bacteria and the constraints on antibiotic use due to their side effects, researchers are exploring potential alternatives [3, 4]. Living microorganisms, such as probiotic bacteria like lactobacilli, play a pivotal role in human health and constitute the most significant component of both human and animal gut microflora [5]. The probiotic microorganisms can inhibit pathogenic organisms' growth and pathogenicity. *Lactobacillus* species are known to generate a diverse range of antimicrobial agents, such as bacteriocins, lactic acids, acetic acids, and other substances, which are found in the culture supernatant of these microorganisms [6].

*Acinetobacter baumannii* is the most common and harmful nosocomial pathogen causing human infections globally, particularly in critical care units (ICUs) [7]. Infections induced by *A. baumannii* commonly lead to substantial rates of morbidity and mortality, reaching up to 60%, including ventilator-associated pneumonia (VAP), septicemia, meningitis, endocarditis, urinary tract infection (UTI), keratitis, and ophthalmitis [8]. *A. baumannii* has rapidly progressed from MDR to extremely drug-resistant (XDR) due to its evolving antibiotic resistance [9].

Although prescription drugs like carbapenems and fluoroquinolones still exhibit effectiveness against XDR *A. baumannii*, the minimum inhibitory concentrations (MICs) have increasingly risen, and nearly carbapenems-resistant *A. baumannii* have been documented [10]. Despite this trend, carbapenems remain the preferred antibiotic therapy for *Acinetobacter* infections. The lack of effective antibiotics for *A. baumannii* infection highlights the need for alternative therapies [11].

The extracellular polysaccharide matrices (EPS) that bacteria self-synthesize serve as a protective measure against biofilms [12]. The presence of biofilms introduces various challenges in the medical field, impeding the clinical treatment of persistent infections associated with various indwelling medical devices, as well as diseases connected to chronic and wound-associated illnesses. Research has indicated that the administration of probiotics can assist in the prevention and/or treatment of infections linked to biofilms [13, 14].

It has been discovered that administering probiotics can help prevent and/or treat infections linked to biofilms. The creation of biofilm and bacterial (pathogenic) MDR causes antibiotics to be ineffective in treating infection [15]. Probiotic lactobacilli, including *Lactobacillus rhamnosus*, seem to have strain-specific antibacterial action, though [16]. Due to its significance, the objectives of this work were to identify, evaluate, and isolate potent lactobacilli with wide antibacterial and anti-biofilm activity against drug-resistant *A. baumannii* strains, as well as to identify the mechanism of inhibition of these organisms.

## 2. Materials and Methods

### 2.1. Sample Collection and Identification

*A. baumannii* were collected from clinical samples, over a six-month period (December 2021 to June 2022). The specimens included respiratory, blood, wounds, burns, surgery, urine, and cerebrospinal fluid (CSF) samples obtained from hospitalized patients in three hospitals in the province of Isfahan (Al-Zahra, Amin, and Milad). These samples were cultured on MacConkey agar and blood agar medium (HiMedia, India) and then were incubated for 24 hours at 37°C. Gram staining and biochemical testing were used to identify the pure strains [17].

### 2.2. Antibiotic Susceptibility Testing

The antibiotic resistance patterns of clinical isolates were assessed using the CLSI-recommended disk diffusion technique [16]. In this study, a range of antibiotics were used including trimethoprim-sulfamethoxazole (10 *µ*g), ciprofloxacin (5 *µ*g), cefepime (30 *µ*g), ceftazidime (30 *µ*g), piperacillin-tazobactam (10–100 *µ*g), ampicillin-sulbactam (10 *µ*g), gentamicin (10 *µ*g), amikacin (30 *µ*g), and meropenem (10 *µ*g) (BD, USA). *A. baumannii* ATCC 19606 strains were used as reference strains. Also, a colistin antibiogram was performed using the MIC determination method with the Phoenix BD device.

### 2.3. Isolation and Identification of *Lactobacillus* Strains

Samples were collected from various local sources to obtain 20 probiotic strains from local dairy products, including sheep yogurt, cow yogurt, camel milk, cow's milk, sheep's milk, goat's milk, and native buttermilk from different regions of Isfahan province in Iran, namely, Shahreza, Golpayegan, Semirom, Fereydunshahr, and Najafabad. For bacterial isolation, 1 mL of each dairy sample underwent homogenization, was subsequently suspended in a 2% w/v sodium citrate solution (Merck, Germany), and was then introduced into 10 mL of MRS broth (HiMedia, India). After 48 hours, 0.02 mL of the solution was spread over MRS agar media following a 24-hour incubation at 37°C [18].

Various tests, including Gram staining, catalase testing, growth at temperatures of 15°C and 45°C, production of acid and gas from glucose, ammonia production from arginine, and fermentation of sugars (arabinose, cellobiose, mannitol, mannose, melibiose, raffinose, ribose, salicin, rhamnose, and xylose), were utilized for the identification of the strains [19].

### 2.4. Evaluation of *Lactobacillus* Strains' Antibacterial Activity

Agar well diffusion and broth microdilution tests were performed to detect antibacterial activity of *Lactobacillus* strains.

### 2.5. Agar Well Diffusion Method

As previously mentioned, the cell-free supernatant (CFS) of *Lactobacillus* cultures was extracted and utilized in the agar well diffusion procedure [20]. To assess antibacterial activity, the growth inhibition zones surrounding the wells were measured following a 24-hour incubation at 37°C. These inhibition zones were then compared with those observed in the control group.

### 2.6. Broth Microdilution Assay

To determine the antibacterial activity (MIC and MBC) of CFS of probiotics against clinical isolates of MDR *A. baumannii*, a broth microdilution test was employed in accordance with previous descriptions [20]. In brief, 100 *μ*L of the diluted (½, ¼, 1/8, and 1/16) CFS of *Lactobacillus* was transferred to 96-well plates in the presence of MRS broth medium. A 96-well plate containing the produced suspension (10^8^ CFU/ml) was then cultivated and incubated for 24 h at 37°C. After that, it was cultured on blood agar medium and kept at 37°C for another 24 hours [21]. The positive and negative controls were the wells devoid of bacteria and extract, respectively. By measuring optical density (OD470 nm), MIC and MBC, representing the lowest concentrations of CSF capable of inhibiting the growth of the pathogen and eradicating all pathogenic bacteria, were determined [22].

### 2.7. Time-Kill Test in Cocultures

By coculturing in microtiter plates, the probiotic was tested for its ability to kill the *Acinetobacter* strain after a minimum period of time. *Lactobacillus* and *A. baumannii* isolates were cultivated in trypticase soy broth (TSB) and MRS broth, respectively. Once the *A. baumannii* strain had been diluted to a 0.5 McFarland turbidity, a suspension of the isolates was prepared. Subsequently, a mixture comprising 100 *μ*L of cell-free supernatant (CFS) from a coculture of *Lactobacillus* strains and 100 *μ*L of *A. baumannii* was prepared in a 96-well microtiter plate and incubated for 24 hours at 37°C. To assess the inhibitory or bactericidal effects, aliquots from the coculture suspension were cultured on blood agar at 1-hour intervals (1 h, 2 h, 4 h, 8 h, 12 h, 24 h, and 48 h) and incubated at 37°C for 24 hours [23]. Each experiment was conducted in duplicate and repeated three times.

### 2.8. Assessment of Acid Tolerance of Probiotic

The *Lactobacillus* strains were inoculated into MRS broth and incubated for 48 h at 37°C. Then, the *Lactobacillus* strains were inoculated into PBS (pH1, pH2, and pH3, pH4 (control)) and PBS (pH4 as control) and incubated for 0, 30 min, and 1 hour, at 37°C. After that, MRS agar was spread out to a density of 50 g/ml, and it was incubated at 37°C for 24 h. The vitality of *Lactobacillus* strains exposed to normal conditions and acidic conditions (pH1, pH2, and pH3) was used to measure the acidic tolerance. This experiment was repeated in duplicate.

### 2.9. Anti-Biofilm Effect of Lactobacilli

For this purpose, the microtiter plate test was utilized. Initially, the bacteria were cultured in MRS medium for one day at 37°C. Suspensions (0.5 McFarland standard) from this culture were then inoculated into MRS medium (supplemented with 0.2% sucrose) containing ½, ¼, and 1/8 MICs of the CFS extracts. A volume of 200 *μ*L of this solution was added to each well of a 96-well microplate. The positive and negative controls were represented by wells without microorganisms and without CFS, respectively. The microplate was incubated at 37°C for 24 hours. Subsequently, the wells were emptied, and crystal violet was added, followed by cleaning with 95% ethanol. Finally, the optical density (OD) of crystal violet associated with biofilms was measured at a wavelength of 570 nm. The experiments were performed three times, utilizing *A. baumannii* ATCC 196006 as a positive control and an uninfected medium as a negative control [22].

### 2.10. Determining the Possible Inhibitory Mechanism

The test was performed to determine whether the inhibitory and antimicrobial effects of the CFS of probiotics were due to the presence of organic acids or other mechanisms. Therefore, to probiotics with pH 4, add 4 drops of NaOH (sodium hydroxide) to neutralize (pH 7), and then in a tube of neutral probiotics and in the other tube, the main probiotic supernatant is placed. Agar well diffusion method on the Muller Hinton agar used as previously described. The plates were cultivated with the 0.5 McFarland turbidity (A. baumannii), create four wells with a sterile glass Pasteur pipette and fill two of them with neutral probiotics (pH=7) and two of them with main probiotics (pH=4), then plates were incubated at 37 °C for 24 h. In parallel, the suspension equivalent to 0.5 McFarland turbidity of Acinetobacter (in a volume of 1000 microliter) was poured into a sterile tube with a screw cap, and the same volume of neutral probiotics was added. The tube was then placed in the incubator. After 4 h, 50 microliter of it was cultured on chocolate agar medium at 37°C for 24 h [18].

### 2.11. High-Performance Liquid Chromatography (HPLC)

The identified *Lactobacillus* strains were cultured in MRS broth medium for 72 hours, after which the culture was centrifuged at 10,000 g for 10 minutes. Following centrifugation, the supernatant was separated from the bacterial pellet and then filtered through a 0.25 *µ*m syringe filter.

**Figure 1:**
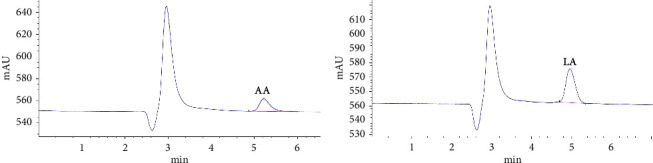
HPLC analysis of organic acids in L. rhamnosus L9. (a) AA acetic acid; (b) LA lactic acid.

To confirm the sterility of the filtrate and absence of *Lactobacilli* growth, a reculturing step in MRS broth medium was conducted for an additional 72 hours. HPLC was carried out using twenty microliters of CFS, employing a flow rate of 1 mL·min^−1^, maintaining pH at 3.6, and utilizing reversed-phase HPLC columns (C18, 25 cm × 4.6 mm) with an aqueous mobile phase (phosphate buffer-CH3CN 10 mM).  Figure 1 shows the (UV) absorbance measured at room temperature at 25°C [24].

### 2.12. MTT Assay

Purchased from the Pasteur Institute in Tehran, Iran, normal subcutaneous connective tissue (L929) cell lines were cultivated in DMEM low glucose medium with 10% FBS and antibiotics (including 50 *μ*g/ml of streptomycin and 50 U/ml of penicillin). The cells were incubated at 37°C with 5% CO2 and 90% humidity. After 24 h, 20 microliter of the probiotic supernatant was filtered twice with a 0.22-micron syringe filter. Then, added and dilution was done in 5 dilutions 1, 0.5, 0.25, 0.125, and 0.062. The microplates were then incubated at 37°C for 24hours. Each research group's cells were incubated at 37°C for 4 h after being exposed to 50 *μ*L of the MTT reagent (5 mg/ml in sterile PBS). To dissolve the formazan crystals, 50 *μ*l of DMSO was added after the culture media had been taken out. The findings were determined using a microplate reader from Bio-Tek Instruments, Inc. of Vermont, USA, at an absorbance of 570 nm [25, 26].

The percentage of cell viability was calculated using

(1) $$\begin{array}{c} \text{Cell viability}\left(\%\right)=\frac{\left(A_{570}\text{ sample}-A_{570}\text{ blank}\right)\times 100}{A_{570}\text{ control}}, \end{array}$$

where A_570_: the absorbance at 570 nm; sample: a monolayer of every cell line plus different treatment concentrations in RPMI media; blank: different treatment concentrations in RPMI medium; and control: a monolayer of every cell line plus RPMI medium with no modifications.

### 2.13. Identification of Selected Lactobacilli

DNA was extracted from pure cultures made from bacterial colonies and stored at −20°C as previously described. By using the universal primers (CinnaGen Co, Iran) [18], the bacteria identified using these traditional tests were verified.

  Universal 1 (27f): AGAGTTTGATCCTGGCTCAG.
  Universal 2 (1492r): TACGGYTACCTTGTTACGACTT.

Using the following steps, the 16S rRNA gene was amplified: initial denaturation at 95°C for 5 minutes, 30 cycles of denaturation at 95°C for 30 seconds, annealing at 54°C for 30 seconds, extension at 72°C for 30 seconds, and a final extension cycle at 72°C for five minutes. Direct sequencing was employed at Bio Magic Gene, Inc., Karaj, Iran, to locate the nucleotide sequences of successful PCR products. The sequences were matched with the NCBI's BLAST search results and then registered.

## 3. Results

### 3.1. *A. baumannii* Isolation and Antibiotic Sensitivity Pattern

During the 6-month period (December 2021 to June 2022) of sample collection, 53 clinical *A. baumannii* strains were obtained and identified from various clinical samples including respiratory secretions (37.7%) (20/53), followed by urine (24.5%) (13/53), blood (18.9%) (10/53), wound (15.1%) (8/53), and CSF (3.8%) (2/53).

Antibiotic susceptibility patterns showed that 6 (11.3%), 43 (81.1%), and 4 (7.5%) strains were MDR, XDR, and PDR, respectively.

**Figure 2:**
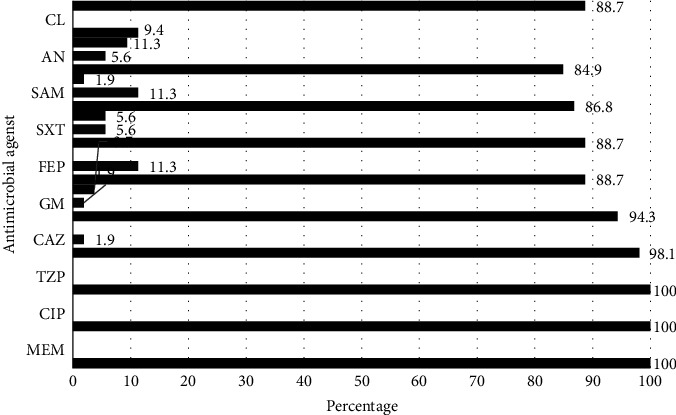
Antibiotic susceptibility pattern of *A. baumannii* isolates. SXT: trimethoprim-sulfamethoxazole; CIP: ciprofloxacin; MEM: meropenem; TZP: piperacillin-tazobactam; AN: amikacin; GM: gentamicin; SAM: ampicillin-sulbactam; CAZ: ceftazidime; FEP: cefepime; CL: colistin.

The most effective antibiotic against them was colistin with only 11.3% resistance, while high percentages of resistance were observed against meropenem (100%), piperacillin/tazobactam (100%), and ciprofloxacin (100%). The full antibiotic susceptibility pattern of *A. baumannii* isolates is presented in Figure 2.

### 3.2. Probiotics Isolation and Identification

Based on our findings, 20 *Lactobacillus* strains were identified from local yogurt and milk samples, and their antimicrobial properties against strains of *A. baumannii* were taken into account. Out of 20 *Lactobacillus* strains, the CFS of one strain (L9) demonstrated an inhibitory effect (showing inhibition zones with a diameter of 14 mm) against every strain of *A. baumannii* in the well diffusion method, according to the results. For this reason, in the remaining trials, the CFSs of the L9 strain were employed.

After 24 hours of incubation, the results showed that L9 was able to grow at pH 1, pH 2, and pH 3 after zero, 30 minutes, and one hour despite variations in the degree of viability. In this test, pH 4 is considered as a positive test.

### 3.3. Antimicrobial Effect of Probiotics against *A. baumannii*

Using the broth microdilution method, it was shown that the MICs and MBCs of CFS extracts of L9 strains against *A. baumannii* strains were both ¼ mg/mL and the ratio of the concentration of MIC and MBC was similar.

**Figure 3:**
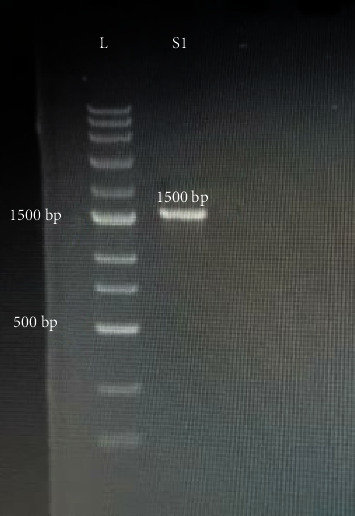
The PCR product of *L. rhamnosus*. L: ladder; S1: sample 1.

Furthermore, no change in OD was observed in the findings of the liquid coculture assay, and after 24 h, the isolates of *A. baumannii* demonstrated 100% suppression of growth. This demonstrated that all *A. baumannii* growth was suppressed by L9 *Lactobacillus* strains. Additionally, when *A. baumannii* strains were inoculated and cultured in blood agar medium, no growth of those strains was seen as compared to positive controls of *A. baumannii* strains without lactobacilli coculture (the killing effect was 100%). Moreover, the minimum time required to inhibit *A. baumannii* strains is only one hour by probiotics, which shows the potential properties of probiotics. Also, L9 strains exhibited tolerance to acid (pH 1, 2, and 3) after 0, 30 min, and 1 hour, respectively (Figure 3).

In addition to Gram staining and biochemical tests, the analysis of phylogenetic relationships based on 16S rDNA indicated a close relationship of both *Lactobacillus* strains to *L. rhamnosus*. The 16S rRNA sequences employed in this study have been assigned NCBI GenBank accession numbers OK637289 (https://www.ncbi.nlm.nih.gov/nuccore/OK637289.1/) and OK637331 (https://www.ncbi.nlm.nih.gov/nuccore/OK637331).

### 3.4. Antibiotic Susceptibility

Among the antibiotics tested, the L9 strain was sensitive to ampicillin, penicillin, tetracycline, linezolid, rifampin, erythromycin, ciprofloxacin, clindamycin, nitrofurantoin, and gentamicin and resistant to teicoplanin, trimethoprim, and vancomycin.

### 3.5. Anti-Biofilm Activity of Probiotic

**Figure 4:**
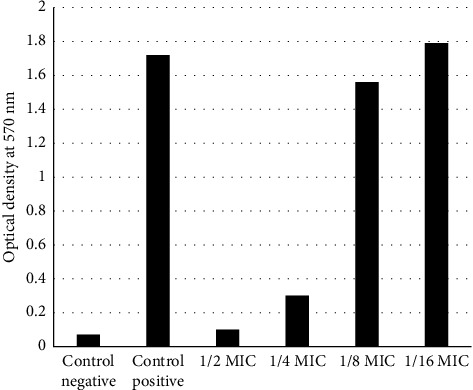
Eradication of *A. baumannii* biofilm in ½, ¼, and 1/8 MICs of the *L. rhamnosus* CFSs.

The outcome of the probiotics' anti-biofilm inhibitory effect demonstrated that, at concentrations of 1, ½, and ¼, the chosen probiotic was able to inhibit biofilm formation and prevent the formation of *A. baumannii* strains; however, at concentrations of 1/8 and 1/16, *A. baumannii* strains formed biofilm, and the probiotic strain was unable to inhibit them (Figure 4).

Investigation of probable inhibition mechanism of L9 strains showed that in contrast to pH 7, in pH 4, the CSF of strain L9 had inhibition zones against *A. baumannii* isolates in the well diffusion method. On the other hand, neutralized supernatants (pH 7) of L9 strains did not have any inhibitory activity against *A. baumannii*, which showed that the inhibitory effects of the L9 strains were due to their organic acid production.

Through the use of HPLC, an assessment of the variety and amount of organic acids generated by different *Lactobacillus* strains revealed that lactic acid and acetic acid were the dominant organic acids produced by all strains. The concentrations of lactic acid and acetic acid were 3.2 gr/ml and 0.5 gr/ml, respectively (Figure 1).

The cytotoxicity test against the L929 fibroblast cell line showed that cell viability of L929 fibroblast cell following treatment with serial concentrations of CFSs for 24 h was 100%. These results were not different from the control bacteria sample (100%).

## 4. Discussion

Globally, MDR-*A. baumannii* strains causing nosocomial infections lead to a growing concern due to high mortality rates and limited treatment options [27, 28]. Most of their important features include survivability in the hospital environment and rapid resistance to various antibiotics. Therefore, alternative treatment methods, such as probiotics, are sought to prevent or treat nosocomial infections [29].

In the present study, *A. baumannii* strains were isolated from various clinical samples to evaluate their antibiotic susceptibility patterns. Additionally, different probiotic strains were isolated from local dairy products to identify a strain with effective antimicrobial activity against *Acinetobacter* strains and investigate their probiotic benefits.

In this study, a large number of *A. baumannii* strains were isolated from various clinical samples, including blood, respiratory secretions, wounds, and urine, respectively, which almost corresponds to studies conducted until now [30, 31]. The results of this study, along with other investigations on the isolation of *A. baumannii* strains, suggest a higher prevalence of this bacterium in respiratory secretions, blood, wounds, and urine samples.

In the current study, the highest sensitivity rate was reported for colistin (88.7%) while ampicillin-sulbactam (1.9%) and gentamicin (3.7%) showed the least susceptibility. The highest resistance was observed for meropenem, ciprofloxacin, and piperacillin-tazobactam antibiotics. This study highlights colistin as the most effective antibiotic. Consistent with results from studies in Iran*, A. baumannii* strains demonstrate significant resistance to currently prescribed antibiotics [32–35].

In a study conducted in Mashhad [16], all isolates were MDR, with imipenem, meropenem, ceftazidime, ceftriaxone, cefixime, cefotaxime, and cephalexin showing the greatest level of resistance (100%) among the isolates. Conversely, the least resistance (19.4%) was found against polymyxin B. This study's outcomes support our conclusions [16]. Similarly, in line with the present study, Nouri et al. and Azimi et al. presented evidence of low sensitivity to meropenem and imipenem in *A. baumannii* isolates, which were resistant to these antibiotics to a large extent [36, 37].

An Iranian meta-analysis examined antibiotic resistance patterns among isolates of *A. baumannii* from ICU-hospitalized patients. The results revealed that the most antibiotic resistance was observed to ceftazidime, ceftriaxone, cefixime, cefotaxime, imipenem, and meropenem, while colistin and polymyxin B exhibited the highest sensitivity. The results of the present study are in agreement with this review and meta-analysis [38].

Contrary to our results regarding the high resistance of the isolates to piperacillin-tazobactam, Azimi et al. reported high sensitivity to this antibiotic [37, 38]. The discrepant results of this study with the mentioned studies can be attributed to the type of examined samples, the use of different antibiotic disks, and variations in geographical locations. Additionally, the overuse of this antibiotic in hospitals could explain the discrepancy.

Lactic acid-producing bacteria are probiotics with anti-inflammatory and antibacterial properties that have been mostly studied in various fields. Lactobacilli, belonging to the Lactobacillaceae family, are the most important and frequently studied microorganisms within this family, encompassing numerous identified species [39].

The results of the agar well diffusion method showed that only one probiotic isolated from local yogurt could create growth inhibition zones on all *A. baumannii* strains. Molecular studies confirmed the identification of our selected probiotic as *L. rhamnosus*, determined through biochemical and molecular PCR tests. In this research, *L. rhamnosus* exhibited a remarkably broad-spectrum activity with inhibitory and lethal effects on all strains of the *A. baumannii* pathogen. The examined probiotic demonstrated a highly effective inhibitory impact in the microtiter plate and coculture method and in determining MIC and MBC. Notably, the MIC and MBC values were equal to ¼ concentration after the coculture of this probiotic bacterium with *Acinetobacter* strains. This means that the probiotic could inhibit and, additionally, kill the pathogen at this concentration, indicating the potential value of *L. rhamnosus*. The results of the lethal time test also showed that the studied probiotic could kill the *Acinetobacter* strains only after one hour, which is a valuable result.

Probiotic bacteria have beneficial antibacterial effects through biofilm formation [40]. This study evidenced the strong anti-biofilm effects of *L. rhamnosus*, effectively preventing replacement, adhesion, and biofilm development by *Acinetobacter* strains through its own biofilm formation. The inhibitory effect of probiotics on biofilm formation by pathogenic strains has been investigated in numerous research studies.

In a research conducted in India, it was documented that *Lactobacillus gasseri*, obtained from the feces of infants, exhibited antagonistic and antimicrobial properties against pathogenic bacteria such as *Staphylococcus aureus*, *Enterococcus faecium*, *Enterobacter*, *Klebsiella pneumoniae*, *A. baumannii*, and *Pseudomonas aeruginosa* [41].

Sultan Dalal et al. reported the antagonistic activity of *L. plantarum* and *L. fermentum* isolated from the feces of healthy infants, against nosocomial infections caused by *A. baumannii* and *P. aeruginosa* [42]. The antimicrobial effect of *L. plantarum* and *L. piscium* strains isolated from goat milk against *A. baumannii* was reported by Fezoni et al. [43]. According to the promising antimicrobial effects of these probiotics, goat milk could be used as an adjuvant treatment for these infections [43].

In a study conducted in the United States [44], topical treatment with the supernatants of *Lactobacillus acidophilus*, *L. casei*, and *L. reuteri* was used on mouse wounds infected with *A. baumannii*. The results showed that the antimicrobial and anti-inflammatory effects of probiotics with local treatment could enhance wound healing [44]. The study, in general, demonstrated that the topical application of some *Lactobacillus* species can be effective against the Gram-negative pathogen *A. baumannii*.

Guan et al. conducted a study on the antibacterial properties, specifically against *Bacillus subtilis* and *Salmonella enterica* and an anti-biofilm of *L. rhamnosus*. The antibacterial activity of *L. rhamnosus* cells was different under different culture conditions, and the intensity of antibacterial effects was found to be unrelated to the biomass. Additionally, an isolated cell-surface extract revealed a wide spectrum of antimicrobial and anti-biofilm capacity. The main components of the extract were identified as polysaccharides and proteins. The properties of the extract indicated that it might be a type of biosurfactant [44].

In another study, CFS extracts of *L. casei* and *L. rhamnosus* were found to be exhibiting antagonistic and anti-biofilm effects against *S. aureus*. This finding suggests that future research could lead to the development of drugs derived from these CFSs to combat *S. aureus* infections [45].

The use of probiotics and their metabolites presents a promising strategy to prevent biofilm growth by various pathogenic microorganisms. In the present study, in addition to the appropriate antimicrobial and anti-biofilm properties of the *L. rhamnosus* strain, other investigated properties include the bile-esculin test, 6.5% salt tolerance, catalase, DNase, hemolysis, CAMP, and cold enrichment, as well as the acid resistance assay and MTT test.

A negative 6.5% salt tolerance test was reported in the current study, consistent with de Vries et al. who reported a low growth of *Lactobacilli* in a salt concentration of ≥5%, and the survival percentage decreased at high salt concentrations [46].

The inhibitory mechanism of the *L. rhamnosus* strain was evaluated in this investigation. To confirm the presence of organic acids in *L. rhamnosus*, the potential inhibitory mechanism was initially determined.

The results showed that the probiotic's inhibitory activity of the probiotic was due to the presence of strong organic acids. In another step, the presence of acetic acid and lactic acid and their concentrations were evaluated using HPLC. The analysis of organic acids in the *L. rhamnosus* supernatant revealed levels of 0.5 g/l for acetic acid and 3.2 g/l for lactic acid. These results strongly suggested that the inhibitory activity of *L. rhamnosus* was primarily due to the presence of organic acids, namely, acetic acid and lactic acid.

## 5. Conclusion

In this study, the probiotic strain isolated from local yogurt showed potential anti-biofilm and antimicrobial properties against all drug-resistant *Acinetobacter* isolates. Given the increasing interest in probiotic microorganisms due to their significant health benefits, further studies are recommended on the mechanisms of action between probiotics and *A. baumannii* strains to find new solutions for biological control and treatment of these infections without the use of antibiotics.

## Data Availability

Data are available on request from the authors.
